# Effects of pedicle screw number and insertion depth on radiographic and functional outcomes in lumbar vertebral fracture

**DOI:** 10.1186/s13018-020-02111-9

**Published:** 2020-12-01

**Authors:** Hao Liu, Weikai Chen, Junxin Zhang, Xiaomin Jiang, Huilin Yang, Rui Qu, Tao Liu

**Affiliations:** 1 Department of Orthopaedic Surgery, The First Affiliated Hospital of Soochow University, 899 Pinghai Road, Suzhou, 215006 Jiangsu China; 2 Department of General Practice, The First Affiliated Hospital of Soochow University, 899 Pinghai Road, Suzhou, 215006 Jiangsu China; 3 The Affiliated Suzhou Hospital of Nanjing Medical University, 26 Daoqian Street, Suzhou, 215006 Jiangsu China

**Keywords:** Vertebral fracture, Sagittal balance, Lumbar pedicle screw fixation, Insertion depth, Screw number

## Abstract

**Background:**

The influence of pedicle screw number and insertion depth on outcomes of lumbar fixation remains uncertain. The purpose of this study was to compare the imaging balance stability and clinical functional improvement of lumbar fracture patients with different pedicle screw numbers and insertion depths.

**Methods:**

Sixty-five patients undergoing lumbar pedicle screw fixation from January 2016 to January 2018 were enrolled. They were included in long screw (LS) group and short screw (SS) group or 6 screw (6S) group and 4 screw (4S) group. The radiographic outcomes were assessed with lumbar lordosis (LL), segmental lordosis (SL), fractured vertebral lordosis (FL), sacral slope (SS), pelvic incidence (PL), and pelvic tilt (PT). The visual analog scale (VAS) and the Oswestry Disability Index (ODI) score were used for functional assessment. Multiple linear regression was performed to identify the risk factors of FL, SL, and LL correction at the final follow-up.

**Results:**

FL, SL, and LL were significantly different in all matching subgroups to compare long and short screws and in most matching subgroups to compare 6 and 4 screws. The SS, PT, and PI seem to be similar in all subgroups in different periods. Significant differences of VAS and ODI were found between LS and SS in the 4S group and between 4S and 6S in the SS group. Insertion depth, screw number, BMD, age, and preoperative imaging data were significant factors for imaging balance stability correction at the final follow-up.

**Conclusions:**

Long screws and 6 screws showed better fracture vertebral restoration and lumbar spinal sagittal stabilities. The surgery type, age, and BMD are important focus points for the treatment of lumbar vertebral fractures.

## Introduction

Spinal fractures are commonly found in orthopedic traumas. The fracture incidence rate is 5 to 6%, with the highest incidence being thoracolumbar fractures, followed by the neck, lumbar vertebrae, and the least being thoracic vertebrae, often with spinal cord or cauda equina injury. Spinal fractures are more common in young male adults and mostly caused by indirect external force. The impact from a foot landing, when it falls from any height to the thoracolumbar region, creates the external force needed to cause spinal fractures. A few fractures are caused by direct external force such as car accidents or firearm injury. The clinical manifestation is deformity and pain of the spine after trauma, which can often be complicated by spinal cord injury. Severe cases can cause paraplegia and even become life-threatening. Improper compression of simple compression fractures can also cause chronic lower back pain.

For new and moderately aged lumbar vertebrae fractures, pedicle screw placement, reduction, and internal fixation techniques are currently the most commonly used surgical treatment options, resulting from instrumentation constructs with high biomechanical strength offered by three-column fixation. By rigid internal fixation, the height of the injured vertebrae is restored and maintained, and the pedicle screw can be removed after the fractured vertebral body is completely healed.

In recent years, restoration of spinal balance, especially spino-pelvic sagittal balance in patients undergoing lumbar fixation, is of crucial importance [1, 2]. However, only a few studies have reported the effects of pedicle screw insertion for spino-pelvic sagittal balance in lumbar fracture cases. We have found from clinical treatments for lumbar fractures that the length of the pedicle screw inserted in the vertebral body, adjacent to the fractured vertebra, often accounts for 50–90% of the anteroposterior diameter of the vertebral body. Some pedicle screw lengths even reached the anterior wall of the vertebral body. In addition, patients often received 6 or 4 pedicle screw fixations for spinal fracture treatment by minimally invasive or open procedures. We suspect that the length of the lumbar pedicle screw needs to be as long as possible or more than 50% of the anteroposterior diameter of the vertebral body. Whether the depth of the lumbar pedicle screw insertion or number of pedicle screw affects the stability of the fractured vertebra and the sagittal balance of the lumbar vertebrae is of question. Therefore, this study aims to compare the imaging balance stability and clinical functional improvement of lumbar fracture patients with different pedicle screw insertion depths and different number of inserted screws.

## Materials and methods

### Patients

Sixty-five patients receiving a posterior lumbar open reduction and a pedicle screw internal fixation operation from January 2016 to January 2018 were enrolled in this retrospective study. Long screw group (LS group): 36 patients underwent long pedicle screw fixation (the leading edge of the screw exceeded 80% of the anteroposterior diameter of vertebral body). Short screw group (SS group): 29 patients underwent short pedicle screw fixation (the leading edge of the screw was less than 60% of the anteroposterior diameter of vertebral body). Six screw group (6S group): 40 patients underwent 6 pedicle screw fixations with two additional screws at the fracture vertebra. Four screw group (4S group): 25 patients underwent 4 pedicle screw fixations without two additional screws at the fracture vertebra. For a comparison between long and short screws, we divided relevant data into 3 matching subgroups (LS and SS in all patients, LS and SS in the 4S group, LS and SS in the 6S group) to analyze the differences. For a comparison between 4 and 6 screws, we divided relevant data into 3 additional matching subgroups (4S and 6S in all patient, 4S and 6S in the LS group, 4S and 6S in the SS group) to analyze the differences. The bone mineral density (BMD) was evaluated using the dual-energy X-ray absorptiometry (DEXA) (Discovery Wi, Hologic, America).

Inclusion criteria were as follows: (1) patients had a trauma-induced single level lumbar (L1-L5) type A1-A3 compressive or a burst fracture according to Magerl classification; (2) patients received posterior short-segment pedicle screw fixation from Medtronic Spine System, including the superior and inferior segment with or without two additional screws at the fracture vertebra; (3) no spinal canal decompression; (4) the follow-up duration was no less than 1 year and the follow-up was held at the same designated hospital. Exclusion criteria were as follows: (1) previous fractures or surgical interventions at the spinal alignment, (2) pathological spinal fractures, (3) symptoms of nerve injury or paralysis caused by the fracture, (4) patients with no valid follow-up information.

### Surgical procedure

Patients were placed in the prone position after general anesthesia with the pelvis and manubrium supported by pads.

For the 6 screw group, an open posterior midline approach was performed, centering the fractured vertebra and systematically revealing the posterior lumbar anatomical structure. After puncturing, placing a guide pin, and measuring the depth, the six pedicle screws were inserted into the fractured vertebra, the adjacent cephalad, and the caudal vertebra. Following the insertion of 6 bent rods, sequential distraction and restoration of lordosis was performed between the ipsilateral adjacent screws in order to reduce kyphosis. When the vertebral height was sufficiently restored, the bent rods were substituted with straight rods, and the free nuts were finally tightened.

For the 4 screw group, a minimally invasive pedicle screw insertion technique by O-arm and stealth navigation (Metromic Navigation, Louisville, CO) was performed. After midline minimal exposure, a reference frame was rigidly attached to the spinous process above surgical level. Based on the device placed on the spinous process, intraoperative CT (O-arm) was used to scan the surgical field. Then, the 3D image data were automatically transferred to the Stealth Station Navigation system and were visualized on a monitor. According to the marked line previously located, a minimal incision was performed. The appropriate depth and trajectory of the pedicle screw insertion was confirmed by using a navigated probe displaying real-time 3 plane images on the monitor. A rigid navigated electric drill, followed by the probe, was used to create a limited pilot canal. Lastly, the 4 pedicle screws were inserted into the superior and the inferior segment properly.

### Radiographic evaluation

**Fig. 1:**
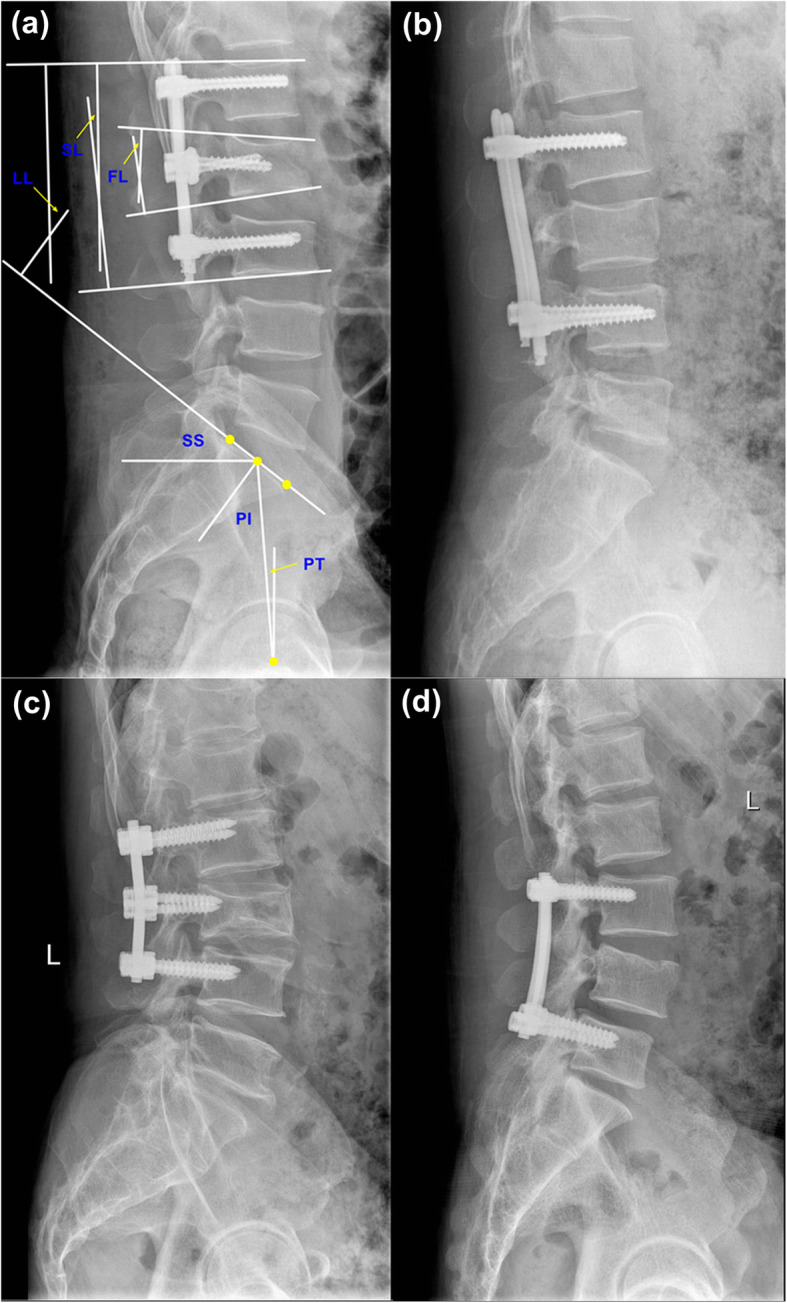
**a** Lumbar pedicle screw fixation with 6 long screws and spino-pelvic sagittal balance measurement. FL, fractured vertebral lordosis; SL, segmental lordosis; LL, lumbar lordosis; SS, sacral slope; PL, pelvic incidence; PT, pelvic tilt. **b** Lumbar pedicle screw fixation with 4 long screws. **c** Lumbar pedicle screw fixation with 6 short screws. **d** Lumbar pedicle screw fixation with 4 short screws

Patients received anterior-posterior and lateral lumbar spinal radiographs in the supine position pre-operatively, in the upright position 1 month after the operation and at the final follow-up. Lumbar lordosis (LL) was defined by Cobb’s method as the angle between the superior endplate of L1 vertebrae and the sacral plate. Segmental lordosis (SL) was also defined by Cobb’s method as the angle between the upper endplate of the superior vertebral body and the lower endplate of the inferior vertebral body. In addition, fractured vertebral lordosis (FL) was measured by Cobb’s method. The sacral slope (SS) was defined as the angle formed between the sacral plate and the horizontal line. The pelvic incidence (PI) was formed by the line perpendicular to the midpoint of the sacral plate and the line between the midpoint of the sacral plate and the centroid of femoral heads. The pelvic tilt (PT) was formed by the angle between the line connecting the midpoint of the sacral plate with the centroid of femoral heads and the vertical line (Fig. 1).

### Clinical assessment

The visual analog scale (VAS) score was used to evaluate patients’ subjective pain perception before surgery, 1 month after surgery, and at the final follow-up (0–10 score, 0 indicated no pain, 10 indicated the most severe pain) [3]. In addition, the Oswestry Disability Index (ODI) score was used to assess the patients’ improvement in quality of life [4].

### Statistical analysis

SPSS 19.0 (IBM, Armonk, NY, USA) was used to process data. Probability values of less than 0.05 indicated a statistically significant difference. For numerical data, the Student’s *t* test was used for data with a normal distribution; otherwise, the Mann–Whitney *U* test was used. Pearson’s Chi-square test and the Fisher exact test were used for categorical data. Multiple linear regression analysis was used to find correlations between various results.

## Results

### Demographics

The demographic data of 6 matching subgroups is shown in Table 1. Sixty-five consecutive patients who received surgical treatment in our hospital and who have completed final follow-up were enrolled into this study. The mean follow-up of LS group was 19.31 ± 4.19 months, and of SS group was 19.90 ± 4.13 months (*P* > 0.05). The mean follow-up of 6S group was 19.15 ± 4.3 months and of 4S group was 20.24 ± 3.87 months (*P* > 0.05). There was no significant difference in the demographic data (gender, age, preoperative BMD, operation segment, and follow-up time) between 6 matching subgroups (*P* > 0.05). All the patients did not complain about any severe complications such as paralysis, nerve root damage, incision infection, or screw breakage.

**Table 1:** The demographic data of the subgroups

	All			6 screws			4 screws		
	Long screws	Short screws	*P*	Long screws	Short screws	*P*	Long screws	Short screws	*P*
Number of patients	36	29	-	22	18	-	14	11	-
Gender (male/female)	15/21	13/16	0.798	9/13	9/9	0.565	6/8	4/7	1.000
Age	48.42 ± 13.32	49.31 ± 10.71	0.771	48.00 ± 13.69	46.56 ± 12.06	0.728	49.07 ± 13.19	53.82 ± 6.16	0.283
BMD	− 1.83 ± 1.23	− 1.98 ± 1.09	0.592	− 1.83 ± 1.29	− 1.64 ± 1.17	0.635	− 1.82 ± 1.19	− 2.55 ± 0.68	0.085
Operation segments	-			-			-		
L1-L3	22	15	0.447	14	11	0.870	8	4	0.302
L2-L4	9	8	0.814	7	3	0.464	2	5	0.177
L3-L5	5	6	0.467	1	4	0.155	4	2	0.661
Follow-up	19.31 ± 4.19	19.90 ± 4.13	0.572	19.09 ± 4.49	19.22 ± 4.18	0.925	19.64 ± 3.82	21.00 ± 3.97	0.395
	All			Long screws			Short screws		
	6 screws	4 screws	*P*	6 screws	4 screws	*P*	6 screws	4 screws	*P*
Number of patients	40	25	-	22	14	-	18	11	-
Gender (male/female)	18/22	10/15	0.692	9/13	6/8	0.908	9/9	4/7	0.702
Age	47.35 ± 12.84	51.16 ± 10.76	0.221	48.00 ± 13.69	49.07 ± 13.19	0.818	46.56 ± 12.06	53.82 ± 6.16	0.076
BMD	− 1.74 ± 1.22	− 2.14 ± 1.05	0.184	− 1.83 ± 1.29	− 1.82 ± 1.19	0.989	− 1.64 ± 1.17	− 2.55 ± 0.68	**0.027**
Operation segments	-			-			-		
L1-L3	25	12	0.251	14	8	0.697	11	4	0.196
L2-L4	10	7	0.789	7	2	0.432	3	5	0.197
L3-L5	5	6	0.311	1	4	0.064	4	2	1.000
Follow-up	19.15 ± 4.30	20.24 ± 3.87	0.306	19.09 ± 4.49	19.64 ± 3.82	0.706	19.22 ± 4.18	21.00 ± 3.97	0.268

Bold represents there is statistical significance between the groups, *p* < 0.05

### Radiographic outcomes

All the radiographic data is shown in Tables 2 and 3. The SL and LL of short screw in 4S group, and the SL and LL of 4 screw in SS group, show no significant difference compared to the preoperative values at the final follow-up (*P* > 0.05). The other FL, SL, and LL at 1 month after surgery and at the final follow-up all showed significant differences (*P* < 0.05) compared with the preoperative values in all subgroups. For the comparison between long and short screws, the FL, SL, and LL of the 3 matching subgroups were significantly different (*P* < 0.05) at the final follow-up but similar at the 1 month after the surgery. The SS, PT, and PI of the 3 matching subgroups were not significantly different (*P* > 0.05) at the two postoperative follow-ups (Table 2). In addition, for the comparison between 4 and 6 screws, the FL, SL, and LL of the 3 matching subgroups were significantly different (*P* < 0.05) at 1 month after surgery and at the final follow-up. The SS, PT, and PI of the 3 matching subgroups were not significantly different (*P* > 0.05) at the two postoperative follow-ups.

**Table 2:** The radiographic data of “long/short screws” groups

	ALL			6 screws			4 screws		
	Long screws	Short screws	*P*	Long screws	Short screws	*P*	Long screws	Short screws	*P*
FL									
Pre	− 17.53 ± 5.74	− 18.62 ± 4.72	0.413	− 17.41 ± 6.75	− 17.83 ± 4.13	0.817	− 17.71 ± 3.87	− 19.91 ± 5.52	0.255
1 month	− 5.72 ± 4.09*	− 7.31 ± 4.29*	0.133	− 4.64 ± 4.51*	− 5.78 ± 2.73*	0.353	− 7.43 ± 2.65*	− 9.82 ± 5.27*	0.152
Final	− 7.94 ± 4.30*	− 11.14 ± 4.36*	**0.004**	− 7.05 ± 4.90*	− 9.83 ± 3.29*	**0.046**	− 9.36 ± 2.73*	− 13.27 ± 5.16*	**0.023**
SL									
Pre	12.78 ± 6.27	11.17 ± 5.24	0.274	13.45 ± 6.76	11.61 ± 4.62	0.332	11.71 ± 5.47	10.45 ± 6.31	0.598
1 month	24.22 ± 4.86*	21.79 ± 5.03*	0.053	25.68 ± 4.75*	23.50 ± 4.34*	0.142	21.93 ± 4.23*	19.09 ± 5.09*	0.141
Final	22.03 ± 5.45*	17.59 ± 6.54*	**0.004**	23.59 ± 5.18*	19.72 ± 5.71*	**0.031**	19.57 ± 5.09*	14.09 ± 6.53	**0.027**
LL									
Pre	31.36 ± 7.27	29.48 ± 7.01	0.297	31.91 ± 7.44	30.33 ± 7.01	0.498	30.50 ± 7.17	28.09 ± 7.12	0.411
1 month	40.81 ± 6.43*	37.62 ± 7.51*	0.070	42.77 ± 5.71*	39.56 ± 7.49*	0.131	37.71 ± 6.45*	34.45 ± 6.71*	0.230
Final	39.47 ± 6.20*	34.59 ± 7.71*	**0.006**	41.18 ± 5.67*	36.94 ± 7.52*	**0.049**	36.79 ± 6.24*	30.73 ± 6.62	**0.028**
SS									
Pre	35.33 ± 8.31	34.79 ± 10.60	0.819	34.86 ± 6.69	35.39 ± 10.15	0.845	36.07 ± 10.62	33.82 ± 11.75	0.620
1 month	34.86 ± 8.35	35.62 ± 10.78	0.750	33.64 ± 6.78	36.83 ± 10.65	0.256	36.79 ± 10.35	33.64 ± 11.19	0.473
Final	36.56 ± 9.36	35.31 ± 10.97	0.623	35.95 ± 7.13	36.50 ± 10.70	0.848	37.50 ± 12.33	33.36 ± 11.65	0.402
PT									
Pre	18.58 ± 8.98	17.97 ± 7.71	0.770	18.36 ± 8.97	17.17 ± 7.79	0.659	18.93 ± 9.34	19.27 ± 7.75	0.922
1 month	16.19 ± 8.24	15.72 ± 6.79	0.806	15.82 ± 8.94	15.11 ± 6.68	0.783	16.79 ± 7.29	16.73 ± 7.18	0.984
Final	15.58 ± 7.65	16.07 ± 6.38	0.785	15.68 ± 8.10	15.39 ± 6.34	0.901	15.43 ± 7.19	17.09 ± 6.50	0.556
PI									
Pre	53.17 ± 13.15	53.41 ± 10.37	0.934	51.86 ± 12.75	53.39 ± 7.16	0.654	55.21 ± 13.98	53.45 ± 14.62	0.762
1 month	51.28 ± 11.63	51.66 ± 9.57	0.889	50.36 ± 11.27	51.89 ± 7.80	0.629	52.71 ± 12.46	51.27 ± 12.36	0.776
Final	51.56 ± 11.77	51.38 ± 9.42	0.948	50.14 ± 11.22	51.28 ± 7.55	0.715	53.79 ± 12.67	51.55 ± 12.32	0.661

*FL* fractured vertebral lordosis, *SL* segmental lordosis, *LL* lumbar lordosis, *SS* sacral slope, *PT* pelvic tilt, *PI* pelvic incidence

*Statistically significant compared with the preoperative, *p* < 0.05; Bold represents there is statistical significance between the groups, *p* < 0.05

**Table 3:** The radiographic data of “6/4 screws” groups

	ALL			Long screws			Short screws		
	6 screws	4 screws	*P*	6 screws	4 screws	*P*	6 screws	4 screws	*P*
FL									
Pre	− 17.60 ± 5.66	− 18.68 ± 4.70	0.428	− 17.41 ± 6.75	− 17.71 ± 3.87	0.879	− 17.83 ± 4.13	− 19.91 ± 5.52	0.258
1 month	− 5.15 ± 3.81*	− 8.48 ± 4.10*	**0.001**	− 4.64 ± 4.51*	− 7.43 ± 2.65*	**0.044**	− 5.78 ± 2.73*	− 9.82 ± 5.27*	**0.011**
Final	− 8.30 ± 4.43*	− 11.08 ± 4.37*	**0.016**	− 7.05 ± 4.90*	− 9.36 ± 2.73*	0.117	− 9.83 ± 3.29*	− 13.27 ± 5.16*	**0.037**
SL									
Pre	12.63 ± 5.90	11.16 ± 5.76	0.329	13.45 ± 6.76	11.71 ± 5.47	0.425	11.61 ± 4.62	10.45 ± 6.31	0.574
1 month	24.70 ± 4.65*	20.68 ± 4.75*	**0.001**	25.68 ± 4.75*	21.93 ± 4.23*	**0.022**	23.50 ± 4.34*	19.09 ± 5.09*	**0.019**
Final	21.85 ± 5.70*	17.16 ± 6.29*	**0.003**	23.59 ± 5.18*	19.57 ± 5.09*	**0.029**	19.72 ± 5.71*	14.09 ± 6.53	**0.021**
LL									
Pre	31.20 ± 7.20	29.44 ± 7.10	0.339	31.91 ± 7.44	30.50 ± 7.17	0.578	30.33 ± 7.01	28.09 ± 7.12	0.413
1 month	41.33 ± 6.68*	36.28 ± 6.64*	**0.004**	42.77 ± 5.71*	37.71 ± 6.45*	**0.019**	39.56 ± 7.49*	34.45 ± 6.71*	0.075
Final	39.28 ± 6.82*	34.12 ± 6.98*	**0.005**	41.18 ± 5.67*	36.79 ± 6.24*	**0.028**	36.94 ± 7.52*	30.73 ± 6.62	**0.032**
SS									
Pre	35.10 ± 8.31	35.08 ± 10.95	0.993	34.86 ± 6.69	36.07 ± 10.62	0.677	35.39 ± 10.15	33.82 ± 11.75	0.706
1 month	35.08 ± 8.76	35.40 ± 10.62	0.894	33.64 ± 6.78	36.79 ± 10.35	0.276	36.83 ± 10.65	33.64 ± 11.19	0.448
Final	36.20 ± 8.80	35.68 ± 11.97	0.841	35.95 ± 7.13	37.50 ± 12.33	0.636	36.50 ± 10.70	33.36 ± 11.65	0.465
PT									
Pre	17.83 ± 8.38	19.08 ± 8.50	0.561	18.36 ± 8.97	18.93 ± 9.34	0.857	17.17 ± 7.79	19.27 ± 7.75	0.485
1 month	15.50 ± 7.91	16.76 ± 7.09	0.518	15.82 ± 8.94	16.79 ± 7.29	0.737	15.11 ± 6.68	16.73 ± 7.18	0.544
Final	15.55 ± 7.27	16.16 ± 6.80	0.737	15.68 ± 8.10	15.43 ± 7.19	0.925	15.39 ± 6.34	17.09 ± 6.50	0.493
PI									
Pre	52.55 ± 10.51	54.44 ± 13.99	0.537	51.86 ± 12.75	55.21 ± 13.98	0.464	53.39 ± 7.16	53.45 ± 14.62	0.987
1 month	51.05 ± 9.77	52.08 ± 12.18	0.708	50.36 ± 11.27	52.71 ± 12.46	0.562	51.89 ± 7.80	51.27 ± 12.36	0.870
Final	50.65 ± 9.64	52.80 ± 12.31	0.435	50.14 ± 11.22	53.79 ± 12.67	0.372	51.28 ± 7.55	51.55 ± 12.32	0.942

*FL* fractured vertebral lordosis, *SL* segmental lordosis, *LL* lumbar lordosis, *SS* sacral slope, *PT* pelvic tilt, *PI* pelvic incidence

*Statistically significant compared with the preoperative, *p* < 0.05; Bold represents there is statistical significance between the groups, *p* < 0.05

### Functional outcomes

The functional outcomes are shown in Table 4. Compared with the preoperative results, the VAS and ODI 1 month after surgery and at the final follow-up all showed significant differences (*P* < 0.05) in all subgroups. In addition, significant differences of VAS and ODI were found between LS and SS in the 4S group, and 4S and 6S in the SS group at the final follow-up (*P* < 0.05).

**Table 4:** The functional outcomes of the subgroups

	All			6 screws			4 screws		
	Long screws	Short screws	*P*	Long screws	Short screws	*P*	Long screws	Short screws	*P*
VAS									
Pre	7.94 ± 1.01	8.10 ± 1.14	0.555	7.95 ± 1.00	8.22 ± 1.22	0.449	7.93 ± 1.07	7.91 ± 1.04	0.964
1 month	3.69 ± 0.95*	3.62 ± 0.82*	0.742	3.91 ± 1.02*	3.78 ± 0.65*	0.639	3.36 ± 0.74*	3.36 ± 1.03*	0.986
Final	2.44 ± 0.88*	2.38 ± 0.73*	0.749	2.55 ± 0.96*	2.39 ± 0.78*	0.581	2.29 ± 0.73*	2.36 ± 0.67*	0.786
ODI									
Pre	31.39 ± 6.12	32.07 ± 6.77	0.672	30.95 ± 5.48	32.78 ± 7.37	0.375	32.07 ± 7.18	30.91 ± 5.79	0.667
1 month	18.39 ± 4.69*	18.76 ± 4.99*	0.760	18.23 ± 4.61*	19.44 ± 4.78*	0.419	18.64 ± 4.97*	17.64 ± 5.35*	0.632
Final	14.89 ± 3.96*	15.03 ± 4.08*	0.885	15.09 ± 3.77*	15.39 ± 4.17*	0.814	14.57 ± 4.38*	14.45 ± 4.03*	0.946
	All			Long screws			Short screws		
	6 screws	4 screws	*P*	6 screws	4 screws	*P*	6 screws	4 screws	*P*
VAS									
Pre	8.08 ± 1.10	7.92 ± 1.04	0.573	7.95 ± 1.00	7.93 ± 1.07	0.941	8.22 ± 1.22	7.91 ± 1.04	0.485
1 month	3.85 ± 0.86*	3.36 ± 0.86*	**0.029**	3.91 ± 1.02*	3.36 ± 0.74*	0.090	3.78 ± 0.65*	3.36 ± 1.03*	0.192
Final	2.48 ± 0.88*	2.32 ± 0.69*	0.456	2.55 ± 0.96*	2.29 ± 0.73*	0.394	2.39 ± 0.78*	2.36 ± 0.67*	0.930
ODI									
Pre	31.78 ± 6.38	31.56 ± 6.50	0.896	30.95 ± 5.48	32.07 ± 7.18	0.601	32.78 ± 7.37	30.91 ± 5.79	0.481
1 month	18.78 ± 4.67*	18.20 ± 5.06*	0.641	18.23 ± 4.61*	18.64 ± 4.97*	0.800	19.44 ± 4.78*	17.64 ± 5.35*	0.353
Final	15.23 ± 3.91*	14.52 ± 4.14*	0.492	15.09 ± 3.77*	14.57 ± 4.38*	0.707	15.39 ± 4.17*	14.45 ± 4.03*	0.559

*VAS* the visual analogue scale, *ODI* the Oswestry Disability Index

*Statistically significant compared with the preoperative, *p* < 0.05; Bold represents there is statistical significance between the groups, *p* < 0.05

### Risk factors of the correction of FL, SL, and LL

Multiple linear regression was performed to identify the risk factors of FL, SL, and LL correction at the final follow-up after surgery compared with preoperative data (Table 5). Insertion depth of pedicle screws, number of pedicle screws, age, gender, BMD, preoperative FL, SL, LL, SS, PT, PI, VAS, and ODI were all included as variables in the analysis. The multiple linear regression showed that the insertion depth (standardized coefficient = 2.219, *P* < 0.001), screw number (standardized coefficient = 1.444, *P* < 0.001), BMD (standardized coefficient = 0.731, *P* < 0.05), and preoperative FL (standardized coefficient = − 0.199, *P* < 0.05) were significant factors for FL correction at the final follow-up. In addition, insertion depth (standardized coefficient = 3.155, *P* < 0.001), screw number (standardized coefficient = 3.179, *P* < 0.001), and age (standardized coefficient = − 0.149, *P* < 0.05) were significant factors for SL correction at the final follow-up. The insertion depth (standardized coefficient = 3.029, *P* < 0.001), screw number (standardized coefficient = 2.841, *P* < 0.001), age (standardized coefficient = − 0.979, *P* < 0.05), BMD (standardized coefficient = 1.156, *P* < 0.05), and preoperative LL (standardized coefficient = − 0.111, *P* < 0.05) were significant factors for LL correction at the final follow-up. Other non-statistically significant factors did not affect the correction of FL, SL, and LL.

**Table 5:** Multiple linear regression for risk factors of FL, SL, and LL correction at the final follow-up compared with preoperative

	FL correction		SL correction		LL correction	
	Standardized coefficients	*P* value	Standardized coefficients	*P* value	Standardized coefficients	*P* value
Insertion depth	2.219	**0.000**	3.155	**0.000**	3.029	**0.000**
4 or 6 screws	1.444	**0.000**	3.179	**0.000**	2.841	**0.000**
Age	− 0.014	0.623	− 0.149	**0.011**	− 0.979	**0.040**
Gender	− 0.154	0.614	0.532	0.370	0.645	0.191
BMD	0.731	**0.023**	− 0.074	0.903	1.156	**0.025**
FL pre	− 0.199	**0.002**	0.094	0.440	− 0.007	0.947
RL pre	0.026	0.666	− 0.089	0.444	0.033	0.729
LL pre	0.005	0.872	− 0.042	0.462	− 0.111	**0.022**
SS pre	− 0.008	0.886	− 0.039	0.732	0.023	0.806
PT pre	0.014	0.825	− 0.059	0.620	0.006	0.951
PI pre	− 0.013	0.821	0.067	0.546	0.012	0.900
VAS pre	0.474	0.059	− 0.426	0.374	0.214	0.589
ODI pre	− 0.065	0.121	0.138	0.088	0.022	0.741

Bold represents there is statistical significance, *p* < 0.05

*FL* fractured vertebral lordosis, *SL* segmental lordosis, *LL* lumbar lordosis

## Discussion

Pedicle screw fixation technique has been a common and necessary surgical method for the treatment of spinal diseases, such as spinal fracture and degenerative or traumatic spinal lesion. The surgeon must first identify the access area on the posterior aspect of the vertebra, the screw insertion angles in both sagittal and transverse planes, number of inserted screws, and the screw-inserted depth. Some researchers have revealed that pedicle screw insertion depth, angle, and quantity affect the biomechanical stability of the screw, and the loading and stiffness of spinal-fixated segment [5–7]. However, in clinical practice, the number and depth of inserted screws usually depend on the surgeon’s clinical experience, which may confuse spinal surgeons. Until now, there is no consensus on how many screws and how long the screws should be inserted for the treatment of lumbar fracture.

Several observed cases showed that the inserted screws had variable depths of penetration. Due to excessive mechanical stress, some of these screws had undergone failure in vivo [8]. To evaluate the effect of the inserted screw number and depth on spinal global balance, we analyzed the following key parameters: pelvic incidence (PI), pelvic tilt (PT), sacral slope (SS), and extent of spinal curvatures, especially lumbar lordosis (LL), segment lordosis (SL), and fractured vertebral lordosis (FL).

The pelvic incidence (PI) as a morphological parameter is an individual variable independent of the body’s position, which increases at the age of 4 to about 18 years and does not change further in the adult age [9, 10]. The PI thus defines the position of the pelvis, and all other pelvic parameters (PT, SS) and spinal curvatures adapt accordingly. The standard value for PI is about 53° ± 9° [11]. Since the PI is a constant quantity, PT and SS are position-dependent quantities [9]. The pelvic tilt (PT) is characteristic for pelvic rotation and changes accordingly when the pelvic tilt is increased, whereby the PT decreases with anteversion and increases with retroversion [10]. The standard values of the PT are approximately 13° ± 6 °[12]. Sacral slope is the angle between the cover plate of S1 and the horizontal and is about 41° ± 8°. Since the PI is defined as a fixed quantity, the PT and SS must mutually adapt to fulfill the equation, when increasing the pelvic tilt, sacral slope must become smaller and vice versa [13]. Comparing the six matching subgroups of study, there is no significant difference for the three spino-pelvic sagittal balance parameters of PI, PT, and SS, which reveals that the number and depth of inserted screws do not affect the spino-pelvic sagittal plane of spinal alignment before and after the construction surgery. However, because the inserted screws usually are taken out 1 to 2 years after the surgery, and patients often have a rest or are non-working during the postoperative period, the short follow-up time and lack of daily activity does not truly reveal the effect of number and depth of inserted screws for spino-pelvic sagittal balance. The radiographic data from the long-term supine rest position for patients suffering from lumbar fracture has a great influence on the measure of sagittal parameters [12].

The lumbar lordosis (LL), which is one of the so-called spinal parameters, is on average 46.5° [13, 14]. LL can be further divided into an upper and a lower section. A study by Roussouly et al. showed that the lower region (L4–S1) is about 70% of the total LL, and the upper region of the lumbar spine (L1–L3) has only 30% of the global lumbar lordosis [10, 15]. The extent of LL is dependent on the value of PI. This close relationship is used to gain a rough estimate of lumbar lordosis. The formula for the ideal LL is LL = PI ± 9°. If a higher PI value is present, the SS increases and the lumbar lordosis intensifies. At low PI values, the SS decreases, and the lumbar lordosis does as well. If there is a mismatch between the two parameters, this often leads to a misalignment of the spinal column and a loss of sagittal balance. The radiographic results of this study showed surgery can reconstruct FL, SL, and LL compared with preoperative data in 6 matching subgroups, but the 4 short screw group received the most dissatisfied outcomes at the final follow-up. In addition, the LS group could reconstruct better in the FL, SL, and LL compared with SS group at the final follow-up, which revealed there is better lumbar spinal stabilities and less failure by using long screw fixation. Furthermore, the 6S group shows better fracture vertebral restoration and lumbar spinal stabilities in comparison to the FL, SL, and LL groups in the other 3 matching subgroups. Patients of the 6S group received sufficient distraction reduction and reconstruction of fractured vertebra by re-bending rods and the addition of a middle screw as a leverage point under direct vision. Patients of the 4S group received a reduction of fractured vertebra only by re-bent rods and insufficient distraction using minimally invasive instruments under navigated vision. Different surgical procedures may lead to different radiographic outcomes. Therefore, planning for spinal sagittal balance restoration, especially for FL, SL, and LL, is an important factor to be taken into account [12].

Pain, disability, and reduced quality of life were common complications after spinal corrective fixation surgery. Failed restoration of an adequate LL could result in sagittal imbalance, which may cause chronic postoperative pain. Furthermore, a pelvic incidence-LL mismatch and a lesser sacral tilt were related to poor clinical outcomes [16]. Functional outcomes showed a significant difference for the pain relief category and the functional improvement category postoperatively compared with preoperative status in all groups. Within 6 matching subgroups, the number and depth of inserted screws were found to be patients’ number one complaint (pain and life quality). Patients inserted with 4 short screws gave lower VAS and ODI compared with others. This may show that radiographic difference in LS and SS and 4S and 6S may not affect subjective feelings nor reduce the quality of life for patients suffering the lumbar vertebral fracture operation.

Clinical parameters, such as screw insertion type and depth, and the patient’s age, bone density, and degree of mobility greatly affect the mechanism of the implant’s failure or success. Jendoubi et al. confirmed the significance of bone density in spine fixation procedures. He found that low-density bone characterizing the elderly is found to have most of its pullout strength (~ 65%) lost with age [8]. Anchorage of pedicle screws strongly depend on their implantation depth and thus loosening the screw is easily done on partially embedded screws [6]. Some studies suggest that optimum anchorage is achieved if the implanted screws through the vertebral body is as deep as possible, even reaching its anterior cortical shell [16]. By analyzing for risk factors of the variety of FL, SL, and LL in the final follow-up after surgery, results of multiple linear regression analysis revealed that screw insertion depth and screw number (4S and 6S) both contribute to the radiographic correction of FL, SL, and LL. Age and BMD are partially related within the three parameters. Therefore, the worse bone quality arising from increasing age, decreasing BMD, increasing quality of surgeries, and the depth of inserted screws are important focus points in preoperative preparation and evaluation.

The limitations of this study must be stated. Although we have provided the assumption is what we think is the most likely to explain the difference between radiographic and functional outcomes for 6 matching subgroups, strong evidence was still lacking. Firstly, a larger sample size of patients should be enrolled in this study for more significant statistics. Secondly, a prospective randomized controlled study may be required for research in the future. In addition, due to the bed position required for spinal fracture patients, preoperative radiographic data was just measured by lumbar X-ray from supine position, which influenced the comparison of spino-pelvic sagittal balance. Some images do not contain bilateral femoral heads. In these cases, the position of the femoral head center was estimated by observing the morphology of the acetabulum. This can indeed lead to measurement errors in PI and PT angle. Finally, a longer follow-up period is needed to validate the conclusions of this study.

## Conclusion

LS and 6S groups showed better fracture vertebral restoration and lumbar spinal sagittal stabilities compared with both the SS and 4S groups. The surgery type, age, and BMD are suggested to be important preoperative focus points for the treatment of lumbar vertebral fractures.

## Data Availability

N/A.

## Abbreviations

LS: Long screw
SS: Short screw
4S: 4 screw
6S: 6 screw
BMD: Bone mineral density
LL: Lumbar lordosis
SL: Segmental lordosis
FL: Fractured vertebral lordosis
SS: Sacral slope
PI: Pelvic incidence
PT: Pelvic tilt
VAS: Visual analogue scale
ODI: Oswestry Disability Index
